# The prolonged interval between induction chemotherapy and radiotherapy is associated with poor prognosis in patients with nasopharyngeal carcinoma

**DOI:** 10.1186/s13014-019-1213-4

**Published:** 2019-01-17

**Authors:** Liang Peng, Jin-Qi Liu, Cheng Xu, Xiao-Dan Huang, Ling-Long Tang, Yu-Pei Chen, Ying Sun, Jun Ma

**Affiliations:** Department of Radiation Oncology, State Key Laboratory of Oncology in South China, Collaborative Innovation Center of Cancer Medicine, Guangdong Key Laboratory of Nasopharyngeal Carcinoma Diagnosis and Therapy, Sun Yat-sen University Cancer Center, No. 651 Dongfeng Road East, Guangzhou, 510060 People’s Republic of China

**Keywords:** Nasopharyngeal carcinoma, Interval, Induction chemotherapy, Radiotherapy, Prognosis

## Abstract

**Objectives:**

Induction chemotherapy (IC) now is gaining recognition for the treatment of nasopharyngeal carcinoma (NPC). The current study was conducted to examine the association between prognosis and the interval between IC and radiotherapy (RT) in NPC patients.

**Methods:**

Patients with newly diagnosed, non-metastatic NPC who were treated with IC followed by RT from 2009 to 2012 were identified from an inpatient database. Overall survival (OS), disease-free survival (DFS), distant metastasis-free survival (DMFS) and locoregional recurrence-free survival (LRFS) were compared between those with interval ≤ 30 and >  30 days by Kaplan-Meier and log-rank analyses; Cox modeling was used for multivariable analysis.

**Results:**

A total of 668 patients met inclusion criteria with median follow-up of 64.4 months. Patients were categorized by interval: 608 patients with interval ≤ 30 days, and 60 with interval >  30 days. The 5-year OS, DFS, DMFS and LRFS rates were 86.6, 78.2, 88.0 and 89.8% for patients with interval ≤ 30 days, respectively, and 69.2, 64.5, 71.2 and 85.1% for patients with interval >  30 days, respectively. The prolonged interval was a risk factor for OS, DFS and DMFS with adjusted hazard ratios (95% confidence intervals) were 2.44 (1.48–4.01), 1.99 (1.27–3.11) and 2.62 (1.54–4.47), respectively.

**Conclusions:**

Prolonged interval >  30 days was associated with a significantly higher risk of distant metastasis and death in NPC patients. Efforts should be made to avoid prolonged interval between IC and RT to minimize the risk of treatment failure.

**Electronic supplementary material:**

The online version of this article (10.1186/s13014-019-1213-4) contains supplementary material, which is available to authorized users.

## Background

Nasopharyngeal carcinoma (NPC) is endemic in southern China, where the age-standardized annual incidence was 5–11 cases per 100,000 in endemic provinces, increasing to 10–27 cases in endemic counties [1]. Due to the anatomic constraints and high radio-sensitivity of NPC, radiotherapy (RT) has become the primary curative treatment. For patients with stage I NPC, RT alone had good efficacy, and led to a 5-year overall survival (OS) rate of over 90% [2]. However, for patients with stage II-IV NPC, accounting for over 90% of the newly-diagnosed cases [3], RT combined with chemotherapy (chemoradiotherapy, CRT) was the recommended standard treatment [4].

Thanks to the introduction of intensity modulated radiotherapy (IMRT) that offered improved target conformity and allowed safer dose escalations, locoregional control has improved substantially compared to 2-dimensional RT, and distant metastasis is now the main consequence of treatment failure [5]. Recently, an individual patient data pooled analysis [6] demonstrated that induction chemotherapy (IC), also known as neoadjuvant chemotherapy, may effectively decrease the distant metastasis rate and improve survival. However, the optimal interval between IC and RT remains unclear.

The prognostic effects of the interval between neoadjuvant treatment and definitive treatment have been studied for rectal [7], breast [8], and non-small cell lung [9] cancer. Chen et al. [10] reported that a prolonged wait time (> 4 weeks) between diagnosis and RT may worsen disease-free survival (DFS) rate for NPC patients. However, to the best of our knowledge, there have been no studies regarding the prognostic value of the interval between IC and RT in NPC. Therefore, we conducted this retrospective study to investigate the prognostic effect of the interval between IC and RT in NPC patients who received IC prior to RT. We hypothesized that the longer interval between IC and RT would be associated with worse survival in NPC patients.

## Methods

### Patients

We retrospectively reviewed an inpatient database that included 2191 patients with newly diagnosed, biopsy-proven, non-metastatic NPC treated at Sun Yat-sen University Cancer Center between November 2009 and October 2012. Patients receiving IC before RT were included, while patients without pretreatment plasma Epstein-Barr virus (EBV) DNA data were excluded.

### Pretreatment evaluation and treatment

All patients underwent a comprehensive pretreatment evaluation, including complete history, physical examination, hematology and biochemistry profiles, magnetic resonance imaging (MRI) of the neck and nasopharynx, chest radiography, abdominal.

ultrasonography, and whole-body bone scanning using single photon emission computed tomography (SPECT). Positron emission tomography and computed tomography (PET/CT) was performed when necessary. All patients were restaged according to the 8th edition of the American Joint Committee on Cancer/International Union against Cancer (AJCC/UICC) staging system based on imaging materials and medical records.

The nasopharyngeal and neck tumor volumes of all patients were treated using radical radiotherapy based on IMRT for the entire course. Gross tumor volumes were defined based on MR, CT and PET/CT imaging before IC; target volumes were delineated slice-by-slice on treatment planning CT scans using an individualized delineation protocol [11], in accordance with the International Commission on Radiation Units and Measurements reports 50 and 62. The prescribed doses were 66–72 Gy to the planning target volume (PTV) of the primary gross tumor volume (GTVnx), 64–70 Gy to the PTV of the GTV of involved lymph nodes (GTVnd), 59.4–63 Gy to the PTV of the high-risk clinical target volume (CTV1), and 50.4–56 Gy to the PTV of the low-risk clinical target volume (CTV2) in 28–33 fractions. All targets were treated simultaneously using the simultaneous integrated boost technique.

During the study, institutional guidelines recommended IMRT alone for stage I NPC and IMRT combined with chemotherapy for stage II-IVa NPC. Three regimes of IC were frequently used: cisplatin (80 mg/m^2^) with 5-fluorouracil (750–1000 mg/m^2^ per day for 5 days), cisplatin (75 mg/m^2^) with docetaxel (75 mg/m^2^), and cisplatin (60 mg/m^2^) plus docetaxel (60 mg/m^2^) with 5-fluorouracil (600–750 mg/m^2^ per day for 5 days) every 3 weeks for 2–4 cycles. Concurrent chemotherapy consisted of cisplatin (80–100 mg/m^2^) every 3 weeks for 2–3 cycles or cisplatin (30–40 mg/m^2^) weekly for 5–7 cycles. Adjuvant chemotherapy was less often chosen because of poor compliance. When possible, salvage treatments (intracavitary brachytherapy, surgery, or chemotherapy) were provided for documented relapse or persistent disease.

### Variables and follow-up

The interval between IC and RT was calculated from the last day of the last cycle of IC to the initiation of RT. Analyzed covariates were as follows: tumor factors, including T category, N category, WHO pathology type and pretreatment plasma EBV DNA concentration; host factors, including age, gender and Charlson comorbidity index (CCI); treatment factors, including IC cycles and use or non-use of concurrent chemotherapy. Pretreatment plasma EBV DNA quantification was performed by real-time quantitative polymerase chain reaction assay amplifying the *Bam*HI-W region of the EBV genome [12]. We added 1 to all EBV DNA values and then performed a natural log transformation to generate a new variable, lnDNA. Charlson comorbidity index was calculated based on medical records to assess the comorbidities [13].

The follow-up duration was measured from first day of treatment to the day of last examination or death. Patients were examined at least every 3 months during the first 2 years, then every 6 months for at least 3 years, and annually thereafter until death. The primary endpoint was OS, defined as the time from the initiation of therapy to death from any cause. The secondary endpoints included DFS, defined as the time from initiation of therapy to failure or death from any cause, whichever occurred first. Distant metastasis-free survival (DMFS) was defined as the time from initiation of therapy to first distant failure. Locoregional recurrence-free survival (LRFS) was defined as the time from initiation of therapy to first locoregional failure.

### Statistical analysis

We intended to dichotomize the interval for simplification of the analysis and better interpretation of the results. Because there was no referenced cutoff point reported previously, we categorized the interval into five consecutive groups to explore the relationship between the interval and OS preliminarily, and we then determined a suitable cutoff point. The associations between the dichotomized interval and other binary or nominal variables were tested with Pearson chi-square test or Fisher’s exact test. Independent samples t-test or Wilcoxon rank-sum test were used for continuous and ordinal variables. Actuarial survival rates were estimated using the Kaplan-Meier method, and survival curves were compared using log-rank test. Hazard ratios (HRs) and 95% confidence intervals (CIs) for each covariate were estimated from univariate Cox regression analyses. The multivariate Cox regression model was used to adjust prognostic effects of the interval for other prognostic factors. Covariates were selected by a backward elimination method with removal criterion of 0.2. The interval and selected covariates entered the multivariate analyses. Age and lnDNA were modeled as continuous variables assuming linear correlation with the outcomes, while the interval and other covariates were modeled as binary variables. Heterogeneity of effect size in different subgroups was appraised by I^2^ statistic, and a value > 25% indicated the existence of heterogeneity [14]. SPSS version 22.0 (IBM Corporation, Armonk, NY, USA), and Stata version 12.0 (StataCorp, College Station, TX, USA) were used for all statistical analyses. Two-tailed *P*-values < 0.05 were considered statistically significant.

## Results

Totally, 1901 of the 2191 patients recorded in database received RT combined with chemotherapy, of which 1086 patients received IC before RT. After 418 patients without EBV DNA data were excluded, 668 patients with complete data were included in subsequent analyses (Additional file 1: Figure S1). Baseline characteristics of included and excluded patients are shown in Additional file 2.

The median follow-up duration for the included 668 patients was 64.4 months (range, 4.57–91.5 months). In total, 108 patients died, 158 experienced failure or died, 89 experienced distant failure, and 70 experienced locoregional failure. The relationships among death, distant and locoregional failure is shown in a Venn diagram (Additional file 1: Figure S2); 95/108 patients died of cancer while 13/108 patients died non-cancer-related deaths. Of the 95 cancer-specific deaths, 68 patients experienced distant metastasis and 40 patients experienced locoregional recurrence. We observed that patients with locoregional recurrence were at less risk of death than were patients with distant metastasis, possibly due to the success of locoregional salvage treatments. The 5-year OS, DFS, DMFS and LRFS rates were 85.0, 77.0, 86.5 and 89.4% respectively.

### Cutoff point of the interval

**Fig. 1:**
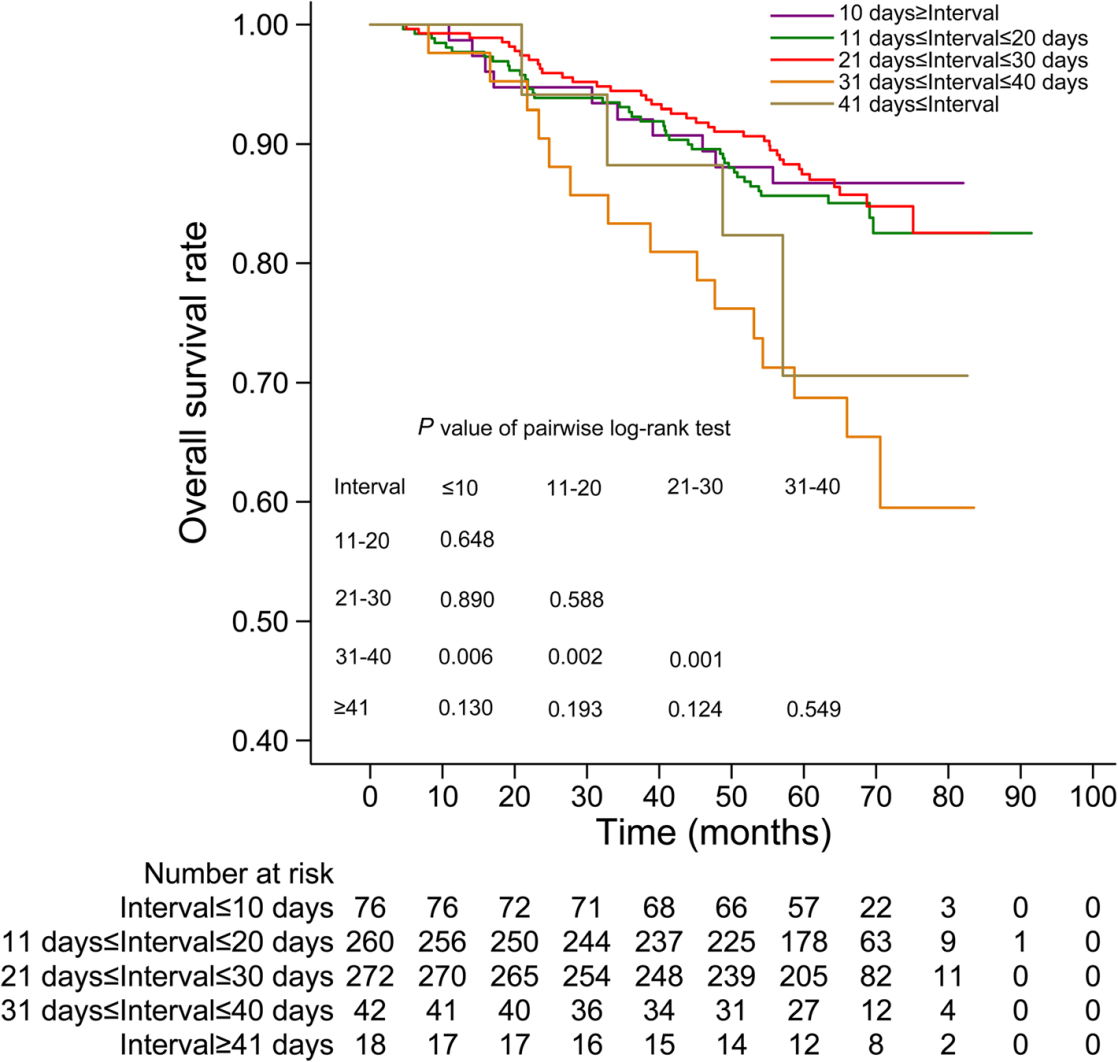
The OS curves based on the interval categorized into five groups. OS, overall survival

The median interval between IC and RT was 20 days (range, 1–67 days; interquartile range, 15–27 days). Three extreme values of 61, 66 and 67 were confirmed by review of medical records. We categorized the interval into ≤10, 11–20, 21–30, 31–40 and ≥ 41 groups, and the 5-year OS rates were 86.7, 85.7, 87.5, 68.7 and 70.6%, respectively. The log rank test showed no significant differences among ≤10, 11–20 and 21–30 groups and between 31 and 40 and ≥ 41 groups (Fig. 1). Therefore, we chose 30 days as the cutoff point to categorize the interval into a ≤ 30 days group with a median of 19 (interquartile range, 14–25) and a >  30 days group with a median of 36 (interquartile range, 32–42).

### Patients characteristics and association with the interval

Patient characteristics for the entire included cohort are displayed in Table 1. Patients with the interval >  30 days tended to receive more cycles of IC (*P* < 0.001) and were less likely to receive concurrent chemotherapy during RT (*P* = 0.028) than were patients with interval ≤ 30 days. We also observed that patients in the > 30 days group were more likely to be at advanced T categories (*P* = 0.034). However, after we recategorized T categories as binary variables (T1–2 and T3–4) before entering the Cox regression, the correlation between T categories and the interval became statistically insignificant (Pearson chi-square test, *P* = 0.154, data not shown). As for the remaining characteristics, no significant correlation between these and the interval was found, even after some of them were recategorized into binary variables (CCI, 0 and ≥ 1; N categories, N0–1 and N2–3; WHO pathology, I/II and III).

**Table 1:** Characteristics of the 668 included patients

Characteristics	Interval ≤ 30 days (608 patients)	Interval > 30 days (60 patients)	*P*-value
Age (years)	44.5 ± 11.0	44.8 ± 11.2	0.808^a^
Gender			0.304^b^
Male	462 (76.0%)	42 (70.0%)	
Female	146 (24.0%)	18 (30.0%)	
CCI score			0.088^c^
0	519 (85.4%)	56 (93.3%)	
1	87 (14.3%)	4 (6.7%)	
≥ 2	2 (0.3%)	0 (0%)	
T category^e^			0.034^c^
T1	54 (8.9%)	3 (5.0%)	
T2	86 (14.1%)	6 (10.0%)	
T3	299 (49.2%)	27 (45.0%)	
T4	169 (27.8%)	24 (40.0%)	
N category^e^			0.211^c^
N0	47 (7.7%)	5 (8.3%)	
N1	339 (55.8%)	29 (48.3%)	
N2	101 (16.6%)	7 (11.7%)	
N3	121 (19.9%)	19 (31.7%)	
Stage^e^			0.105^c^
II	77 (12.7%)	7 (11.7%)	
III	263 (43.3%)	19 (31.7%)	
IVa	268 (44.1%)	34 (56.7%)	
EBV DNA (10^3^ copies/ml)	5.6 (0–3290)	6.1 (0–3660)	0.476^c^
lnDNA^f^	7.5 ± 4.0	7.8 ± 4.1	0.504^a^
WHO pathology			0.728^d^
I	6 (1.0%)	0 (0%)	
II	28 (4.6%)	4 (6.7%)	
III	574 (94.4%)	56 (93.3%)	
IC cycles			< 0.001^c^
1	75 (12.3%)	5 (8.3%)	
2	315 (51.8%)	21 (35.0%)	
3	187 (30.8%)	18 (30.0%)	
≥ 4	31 (5.1%)	16 (26.7%)	
Concurrent chemotherapy			0.028^b^
Yes	497 (81.7%)	42 (70.0%)	
No	111 (18.3%)	18 (30.0%)	

Data presented as number (%), mean ± standard deviation or median (range)

*Abbreviations*: *CCI* Charlson comorbidity index, *EBV* Epstein-Barr virus, *IC* Induction chemotherapy

^a^Independent samples t-test

^b^Pearson chi-square test

^c^Wilcoxon rank-sum test

^d^Fisher’s exact test

^e^According to the 8th edition of AJCC/UICC staging system

^f^lnDNA = ln (EBV DNA × 1000 + 1)

### Prognostic effect of the interval for NPC patients

**Fig. 2:**
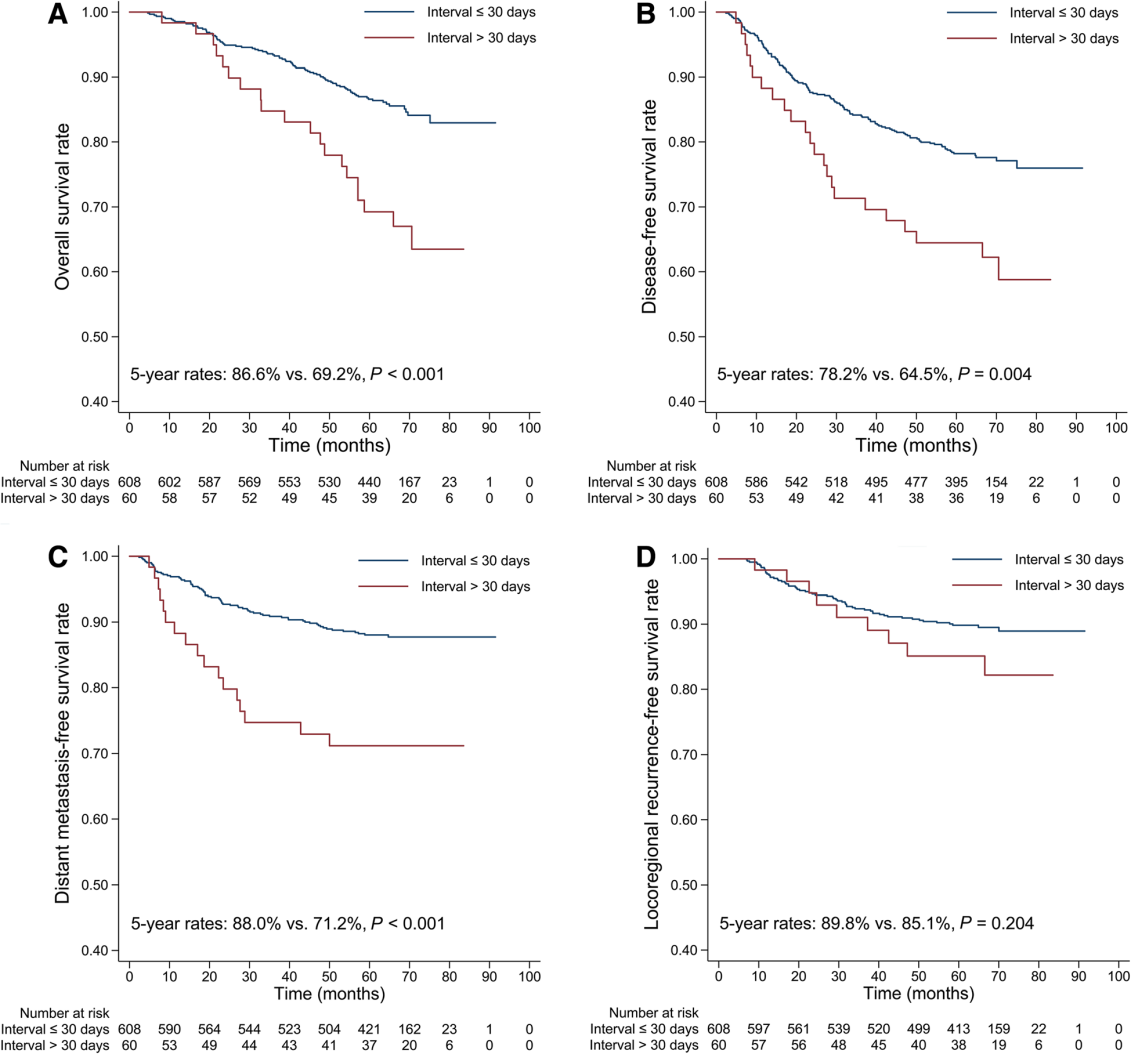
The OS (**a**), DFS (**b**), DMFS(**c**), and LRFS (**d**) curves based on the interval categorized into two groups. OS, overall survival; DFS, disease-free survival; DMFS, distant metastasis-free survival; LRFS, locoregional recurrence-free survival

The 5-year OS rate was significantly lower for patients with interval > 30 days than for those with interval ≤ 30 days (69.2% vs. 86.6%, *P* < 0.001; Fig. 2a). The 5-year DFS rate (64.5% vs. 78.2%, *P* = 0.004; Fig. 2b) and DMFS rate (71.2% vs. 88.0%, *P* < 0.001; Fig. 2c) were also significantly lower for patients with interval > 30 days than for those with interval ≤ 30 days. However, the difference in 5-year LRFS rate (85.1% vs. 89.8%, *P* = 0.204; Fig. 2d) failed to reach significance. The unadjusted HRs and 95% CIs for the interval and other covariates estimated from univariate Cox regression are shown in Table 2.

**Table 2:** Univariate analyses of the interval and other covariates based on Cox regression model

Factors	OS		DFS		DMFS		LRFS	
	HR (95% CI)	P*	HR (95% CI)	P*	HR (95% CI)	P*	HR (95% CI)	P*
Interval between IC and RT (> 30 vs. ≤ 30 days)	2.43 (1.49–3.95)	< 0.001	1.90 (1.22–2.95)	0.005	2.67 (1.57–4.53)	< 0.001	1.57 (0.78–3.16)	0.208
Age (continuous)	1.02 (1.01–1.04)	0.010	1.02 (1.00–1.03)	0.040	1.00 (0.98–1.02)	0.899	1.01 (0.99–1.03)	0.240
Sex (Female vs. Male)	0.85 (0.54–1.34)	0.487	1.00 (0.69–1.43)	0.985	1.04 (0.65–1.67)	0.866	1.24 (0.74–2.07)	0.425
T category (T3–4 vs. T1–2)	1.45 (0.87–2.41)	0.149	1.28 (0.86–1.92)	0.228	1.15 (0.68–1.92)	0.608	1.54 (0.81–2.92)	0.192
N category (N2–3 vs. N0–1)	2.22 (1.52–3.24)	< 0.001	1.66 (1.21–2.28)	0.002	3.17 (2.08–4.85)	< 0.001	0.82 (0.49–1.38)	0.464
lnDNA (continuous)	1.06 (1.01–1.12)	0.022	1.07 (1.02–1.12)	0.004	1.17 (1.09–1.26)	< 0.001	1.04 (0.98–1.11)	0.187
WHO pathology (III vs. I/II)	0.59 (0.30–1.18)	0.135	0.75 (0.41–1.39)	0.361	1.25 (0.46–3.41)	0.661	0.43 (0.21–0.90)	0.025
CCI (≥ 1 vs. 0)	2.03 (1.29–3.21)	0.002	1.74 (1.18–2.57)	0.005	1.22 (0.69–2.16)	0.496	1.38 (0.74–2.58)	0.310
IC cycles (> 2 vs. ≤ 2)	1.29 (0.88–1.89)	0.187	1.25 (0.91–1.71)	0.170	1.39 (0.92–2.12)	0.118	1.26 (0.79–2.03)	0.336
Concurrent chemotherapy (Yes vs. No)	0.97 (0.60–1.56)	0.903	0.99 (0.67–1.46)	0.946	1.10 (0.64–1.89)	0.728	1.05 (0.58–1.92)	0.869

*Abbreviations*: *OS* Overall survival, *DFS* Disease-free survival, *DMFS* Distant metastasis-free survival, *LRFS* Locoregional recurrence-free survival, *HR* Hazard ratio, *CI* Confidence interval, *IC* Induction chemotherapy, *RT* Radiotherapy, *CCI* Charlson comorbidity index

*Wald chi-square test

Results of multivariable analyses are presented in Table 3. The interval > 30 days remained a significant negative prognostic factor for NPC patients in terms of OS (HR 2.44, 95% CI 1.48–4.01), DFS (HR 1.99, 95% CI 1.27–3.11) and DMFS (HR 2.62, 95% CI 1.54–4.47).

**Table 3:** Multivariate analyses of prognostic factors based on Cox regression model

Outcomes	Variables in the final model	HR (95% CI)	P*
OS	Interval (> 30 vs. ≤ 30 days)	2.44 (1.48–4.01)	< 0.001
	N category (N2–3 vs. N0–1)	2.13 (1.44–3.16)	< 0.001
	CCI (≥ 1 vs. 0)	2.21 (1.38–3.54)	0.001
	Age (continuous)	1.02 (1.00–1.04)	0.042
	lnDNA (continuous)	1.04 (0.98–1.10)	0.173
	T category (T3–4 vs. T1–2)	1.42 (0.85–2.36)	0.183
DFS	Interval (> 30 vs. ≤ 30 days)	1.99 (1.27–3.11)	0.003
	CCI (≥ 1 vs. 0)	1.87 (1.26–2.79)	0.002
	N category (N2–3 vs. N0–1)	1.58 (1.14–2.18)	0.006
	lnDNA (continuous)	1.06 (1.01–1.10)	0.018
	Age (continuous)	1.01 (1.00–1.03)	0.126
DMFS	Interval (> 30 vs. ≤ 30 days)	2.62 (1.54–4.47)	< 0.001
	N category (N2–3 vs. N0–1)	3.03 (1.93–4.77)	< 0.001
	lnDNA (continuous)	1.12 (1.04–1.20)	0.002
	CCI (≥ 1 vs. 0)	1.50 (0.84–2.66)	0.171
LRFS	Interval (> 30 vs. ≤ 30 days)	1.53 (0.76–3.08)	0.238
	WHO pathology (III vs. I/II)	0.42 (0.20–0.89)	0.023
	N category (N2–3 vs. N0–1)	0.63 (0.37–1.07)	0.086
	lnDNA (continuous)	1.06 (0.99–1.13)	0.099

Covariates including: age (continuous), gender (female vs. male), CCI score (≥ 1 vs. 0), T category (T3–4 vs. T1–2), N category (N2–3 vs. N0–1), lnDNA (continuous), WHO pathology type (III vs. I/II), IC cycles (> 2 vs. ≤ 2), concurrent chemotherapy (yes vs. no)

*Abbreviations*: *OS* Overall survival, *DFS* Disease-free survival, *DMFS* Distant metastasis-free survival, *LRFS* Locoregional recurrence-free survival, *IC* Induction chemotherapy, *CCI* Charlson comorbidity index, *HR* Hazard ratio, *CI* Confidence interval

*Wald chi-square test

### Subgroup analyses

We further explored the prognostic effects of the interval in subgroups stratified by N category, IC cycles and concurrent chemotherapy. For patients with N2–3 disease, the interval remained a significant prognostic factor in terms of OS, DFS and DMFS. By contrast, the interval may not exert a significant impact on OS, DFS and DMFS for patients with N0–1 disease (Additional file 1: Figure S3). After the cohort was stratified by IC cycles (Additional file 1: Figure S4) or concurrent chemotherapy (Additional file 1: Figure S5), the interval remained a significant prognostic factor, indicating its independence.

Heterogeneity analyses of effect size of the interval in subgroups suggested the existence of a modification effect by N category and concurrent chemotherapy. The interval > 30 days may be more dangerous for patients with advanced N category or not receiving concurrent chemotherapy during RT in terms of OS and DFS. Heterogeneity was not detected in terms of DMFS, possibly due to the lower number of events in subgroups. (Additional file 1: Figure S6)

To help readers gain a general understanding of our study, we provided a study profile recommended by the Reporting Recommendations for Tumor Marker Prognostic Studies (REMARK) [15] in Table 4.

**Table 4:** The REMARK profile

(a) Patients, treatment and variables			
Study and marker	Remarks		
Prognostic factor	M = the interval between IC and RT (days from the end of the last cycle of IC to the initiation of RT); categorized into 5 groups (≤ 10, 11–20, 21–30, 31–40 and ≥ 41), or 2 groups (≤ 30 and > 30)		
Further variables	v1 = age, v2 = gender, v3 = CCI score, v4 = T category, v5 = N category, v6 = lnDNA, v7 = WHO pathology type, v8 = IC cycles, v9 = concurrent chemotherapy		
Patients	Number	Remarks	
Assessed for eligibility	2191	Disease: newly diagnosed, biopsy-proven, non-metastatic NPC Patient source: 2009.11–2012.10, Sun Yat-sen University Cancer Center	
Excluded	1523	Exclusion criteria: not receiving IC before RT; without pretreatment plasma EBV DNA	
Included	668	Treatment: all received IC and IMRT with or without concurrent chemotherapy	
Primary outcome events			
OS	108	OS: from the initiation of therapy to death from any cause	
Secondary outcomes events			
DFS	158	DFS: from the initiation of therapy to failure or death from any cause, whichever occurred first	
DMFS	89	DMFS: from the initiation of therapy to first distant failure	
LRFS	70	LRFS: from the initiation of therapy to first locoregional failure	
(b) Statistical analyses of survival outcomes			
Analysis		Variables considered	Results/remarks
A1: Univariate for OS		M	Fig. 1; the interval was categorized into 5 groups
A2: Univariate for OS, DFS, DMFS, LRFS		M, v1-v9	Fig. 2, Table 2; the interval was categorized into 2 groups
A3: Multivariate for OS, DFS, DMFS, LRFS		M, v1-v9	Table 3; variables in final model: OS (M, v1, v3, v4, v5, v6), DFS (M, v1, v3, v5, v6), DMFS (M, v3, v5, v6), LRFS (M, v5, v6, v7)
A4: Interval in v5 subgroups for OS, DFS, DMFS		M, v5	Additional file 1: Figures S3 and S6
A5: Interval in v8 subgroups for OS, DFS, DMFS		M, v8	Additional file 1: Figures S4 and S6
A6: Interval in v9 subgroups for OS, DFS, DMFS		M, v9	Additional file 1: Figures S5 and S6

*Abbreviations*: *IC* Induction chemotherapy, *RT* Radiotherapy, *CCI* Charlson comorbidity index, *NPC* Nasopharyngeal carcinoma, *EBV* Epstein-Barr virus, *IMRT* Intensity modulated radiotherapy, *OS* Overall survival, *DFS* Disease-free survival, *DMFS* Distant metastasis-free survival, *LRFS* Locoregional recurrence-free survival

## Discussion

As pretreatment plasma EBV DNA is an important prognostic factor for NPC patients, especially in predicting the distant metastasis [16], we excluded patients without EBV DNA data to minimize the potential confounding bias. Since the distribution of clinicopathologic factors (age, gender, T category, N category, overall stage and WHO pathology type) between the included cohort and the excluded cohort without EBV DNA data was statistically equivalent, the selection bias seemed to be not existent.

Both univariate and multivariate analyses revealed that prolonged interval > 30 days between IC and RT was a negative prognostic factor for NPC compared to the interval ≤ 30 days, with 2.44-fold increased risk of death, 1.99-fold increased the risk of failure or death, and 2.62-fold increased risk of distant failure. However, the interval was not associated with the risk of locoregional failure. Considering information of the Venn diagram (Additional file 1: Figure S2), we could infer that the worse OS and DFS rates associated with prolonged interval were caused by the worse DMFS, when the LRFS remained unchanged due to good locoregional control of IMRT.

The reason for an association between the interval and prognosis of NPC patients is likely complex and multifactorial. In multivariate analyses, we set a loose criterion for selecting covariates to avoid neglecting the potential important prognostic factors and to better adjust for effects of the interval. In the cohort, we found that the interval was associated with two other treatment factors, IC cycles and concurrent chemotherapy, however neither factor was selected into the final multivariate models. Considering this, we further conducted subgroup analyses and confirmed the independent impact of interval on NPC patients.

A study from Taiwan [17] reported that NPC patients with more comorbidities were associated with a prolonged wait time from diagnosis to RT, however, patients in this cohort did not receive IC before RT. In our cohort, there was no significant association between the interval between IC and RT and comorbidities, possibly due to most patients (666/668) being scored 0 or 1 by CCI, indicating relatively mild comorbidities. We found that comorbidity was an important prognostic factor for NPC patients in terms of OS and DFS, in accordance with study reported by Guo et al. [18]. After adjusting for CCI in the multivariate analyses, the interval remained a prognostic factor with statistical significance in terms of OS, DFS and DMFS.

A possible explanation is that the prolonged interval between IC and RT increased the risk of micro-metastasis from the locoregional lesions. IC was thought to be favorable for eradication the micro-metastasis and shrinking of locoregional lesions [19, 20], however the definitive eradication of locoregional lesions could not be achieved without RT. We postulated that tumor cells may leave locoregional lesions for distant metastasis during the interval when no anti-tumor treatment was used. The longer the interval between IC and RT, the greater the risk of micro-metastasis. However, for patients with early N categories at a low risk of distant metastasis, the interval may not exert impact on prognosis. On subgroup analysis, we also observed that concurrent chemotherapy during RT may weaken the risk posed by prolonged interval, attributable to the advantage of eradicating micro-metastasis by systemic therapy [21]. Chen et al. [10] also reported that NPC patients who did not receive IC before RT may be more vulnerable to distant failure as wait time from diagnosis to RT increased, also indicating that tumor may be more likely to progress during the anti-tumor treatment-free period. However, we believed that there should be a threshold of the interval considering the concept of chemotherapy dose intensity and the characterization of tumor cell cycle kinetics [22]. Therefore, a cutoff point to dichotomize the interval should be appropriate.

We observed that the prolonged interval between IC and RT was often caused by severe chemotherapy-related complications, after the hospital crowding problem was solved by augmentation of RT instruments and optimization of RT process at our institution. Due to the retrospective nature of our study, the IC related complications were difficult to evaluate, and we could not adjust for complications in the multivariate analyses. Though complications may influence the survival of patients, tumor control may not have direct associations with complications per se [23]. A multidisciplinary team approach may help prevent and treat complications and avoid prolonging the interval between IC and RT [24]. Adding another cycle of IC may be a way to avoid prolonged interval in cases where RT was delayed for other unexpected reasons, though Peng et al. [25] thought two cycles of IC were enough for NPC patients.

Our study was limited by its retrospective and single-center nature without external validation of results. Considering that a prospective randomized clinical trial to elucidate the relationship between interval and prognosis in NPC patients may be ethically unacceptable, further prospective or retrospective observational studies based on real-word data from multicenter are needed in the future.

## Conclusion

The prolonged interval > 30 days between IC and RT was associated with a high risk of distant failure, and therefore poor survival prognosis for NPC patients receiving IC before RT, especially for patients with advanced N category. Although confounding factors may underlie this relationship, until a causal relationship can be excluded, efforts should be made to avoid prolonging the interval between IC and RT to minimize risk of distant failure and death.

## Additional files

Additional file 1: **Figure S1.** Flow diagram of selection of included patients. NPC, nasopharyngeal carcinoma; CRT, chemoradiotherapy; IC, induction chemotherapy; EBV, Epstein-Barr virus. **Figure S2.** Distribution of events occurring in 158 patients. **Figure S3.** Subgroup analyses based on N category. Kaplan–Meier survival curves were delineated based on the interval for overall survival (a, b), disease-free survival (c, d), distant metastasis-free survival (e, f) of N0–1 and N2–3 subgroups. **Figure S4.** Subgroup analyses based on IC cycles. Kaplan–Meier survival curves were delineated based on the interval for overall survival (a, b), disease-free survival (c, d), distant metastasis-free survival (e, f) of IC cycles ≤2 and >  2 subgroups. IC, induction chemotherapy. **Figure S5.** Subgroup analyses based on concurrent chemotherapy. Kaplan–Meier survival curves were delineated based on the interval for overall survival (a, b), disease-free survival (c, d), distant metastasis-free survival (e, f) of concurrent chemotherapy and no concurrent chemotherapy subgroups. **Figure S6.** Results of subgroup analyses summarized in forest plot. The HR (95% CI) of the interval for OS, DFS and DMFS in different subgroups and heterogeneity of HR between relative subgroups were shown. HR, hazard ratio; CI, confidence interval; OS, overall survival; DFS, disease-free survival; DMFS, distant metastasis-free survival; CC, concurrent chemotherapy. (DOCX 632 kb)

Additional file 2: **Table S1.** Baseline characteristics of included and excluded patients. (DOCX 19 kb)

## Abbreviations

AJCC/UICC: American Joint Committee on Cancer/International Union against Cancer
CCI: Charlson comorbidity index
CI: Confidence interval
CRT: Chemoradiotherapy
CTV: Clinical target volume
DFS: Disease-free survival
DMFS: Distant metastasis-free survival
EBV: Epstein-Barr virus
GTV: Gross tumor volume
HR: Hazard ratio
IC: Induction chemotherapy
IMRT: Intensity modulated radiotherapy
LRFS: Locoregional recurrence-free survival
MRI: Magnetic resonance imaging
NPC: Nasopharyngeal carcinoma
OS: Overall survival
PET/CT: Positron emission tomography and computed tomography
PTV: Planning target volume
REMARK: Reporting Recommendations for Tumor Marker Prognostic Studies
RT: Radiotherapy
SPECT: Single photon emission computed tomography
