# Supplementary material for: The prolonged interval between induction chemotherapy and radiotherapy is associated with poor prognosis in patients with nasopharyngeal carcinoma
Source: Radiat Oncol. 2019 Jan 17;14:9. doi: 10.1186/s13014-019-1213-4 (PMC6335732; doi:10.1186/s13014-019-1213-4)
Supplement: Supplementary file 1 — Figure S1. Flow diagram of selection of included patients. NPC, nasopharyngeal carcinoma; CRT, chemoradiotherapy; IC, induction chemotherapy; EBV, Epstein-Barr virus. Figure S2. Distribution of events occurring in 158 patients. Figure S3. Subgroup analyses based on N category. Kaplan–Meier survival curves were delineated based on the interval for overall survival (a, b), disease-free survival (c, d), distant metastasis-free survival (e, f) of N0–1 and N2–3 subgroups. Figure S4. Subgroup analyses based on IC cycles. Kaplan–Meier survival curves were delineated based on the interval for overall survival (a, b), disease-free survival (c, d), distant metastasis-free survival (e, f) of IC cycles ≤2 and > 2 subgroups. IC, induction chemotherapy. Figure S5. Subgroup analyses based on concurrent chemotherapy. Kaplan–Meier survival curves were delineated based on the interval for overall survival (a, b), disease-free survival (c, d), distant metastasis-free survival (e, f) of concurrent chemotherapy and no concurrent chemotherapy subgroups. Figure S6. Results of subgroup analyses summarized in forest plot. The HR (95% CI) of the interval for OS, DFS and DMFS in different subgroups and heterogeneity of HR between relative subgroups were shown. HR, hazard ratio; CI, confidence interval; OS, overall survival; DFS, disease-free survival; DMFS, distant metastasis-free survival; CC, concurrent chemotherapy. (DOCX 632 kb) [file 13014_2019_1213_MOESM1_ESM.docx]

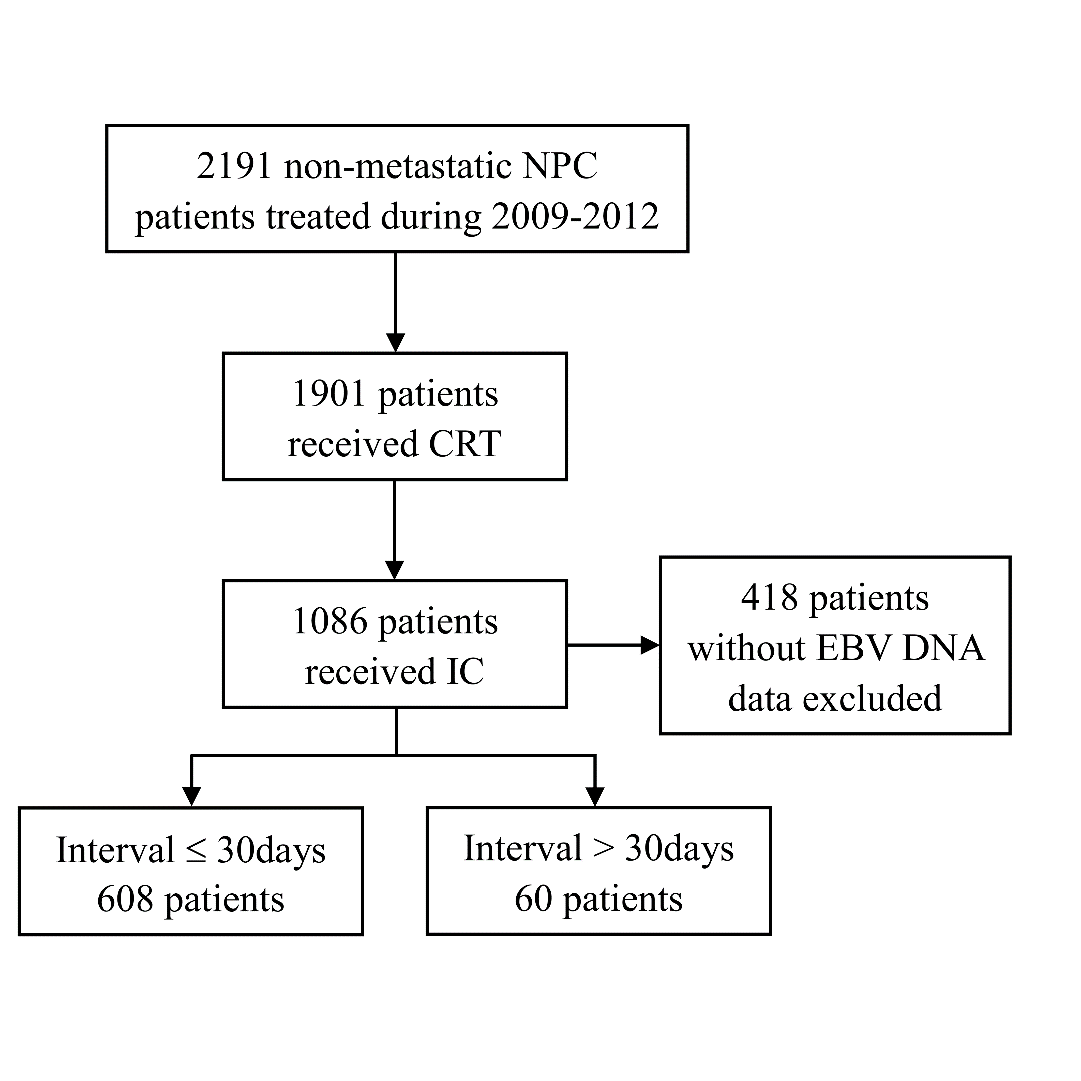


**Figure S1** Flow diagram of selection of included patients. NPC, nasopharyngeal carcinoma; CRT, chemoradiotherapy; IC, induction chemotherapy; EBV, Epstein-Barr virus.


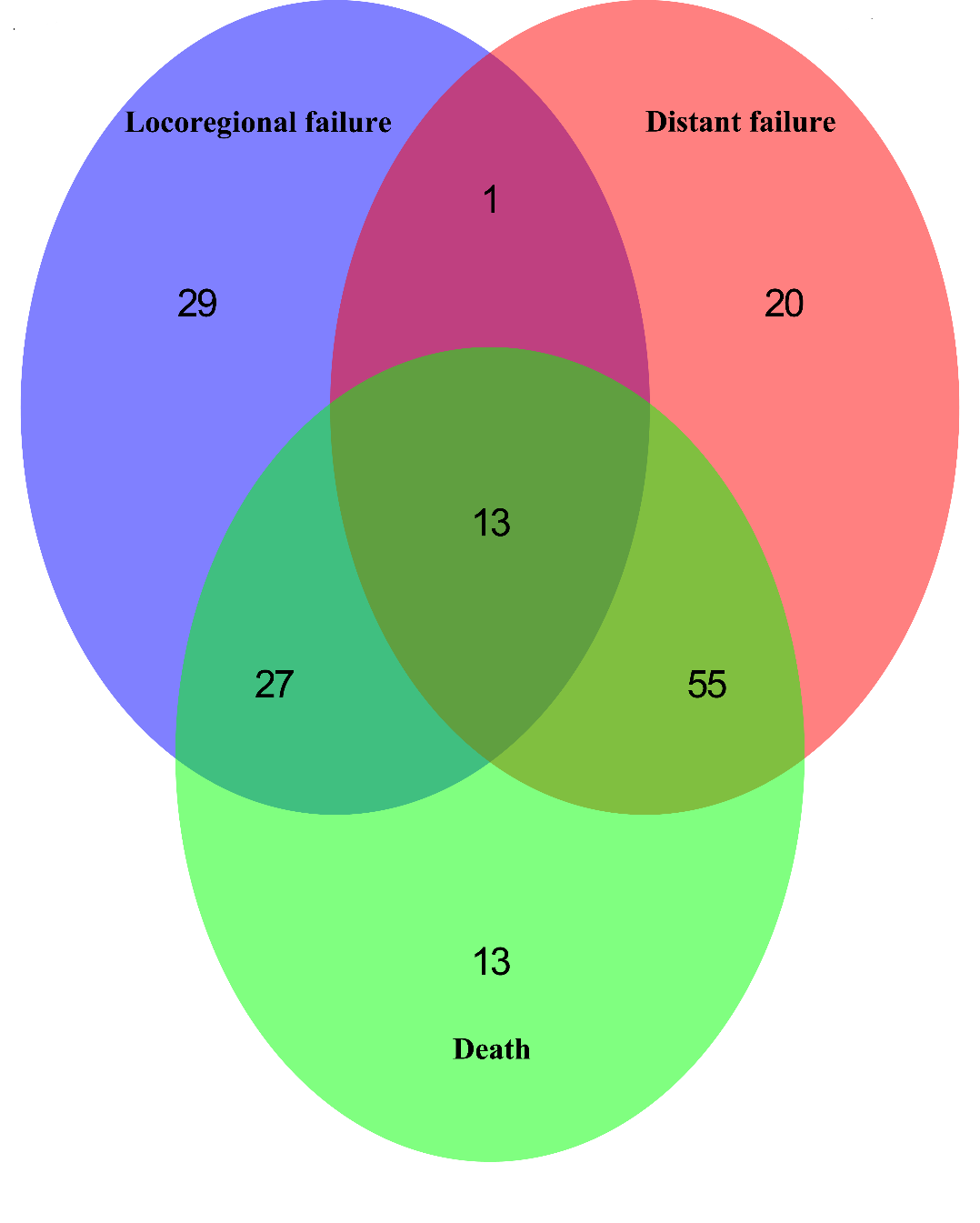


**Figure S2** Distribution of events occurring in 158 patients.


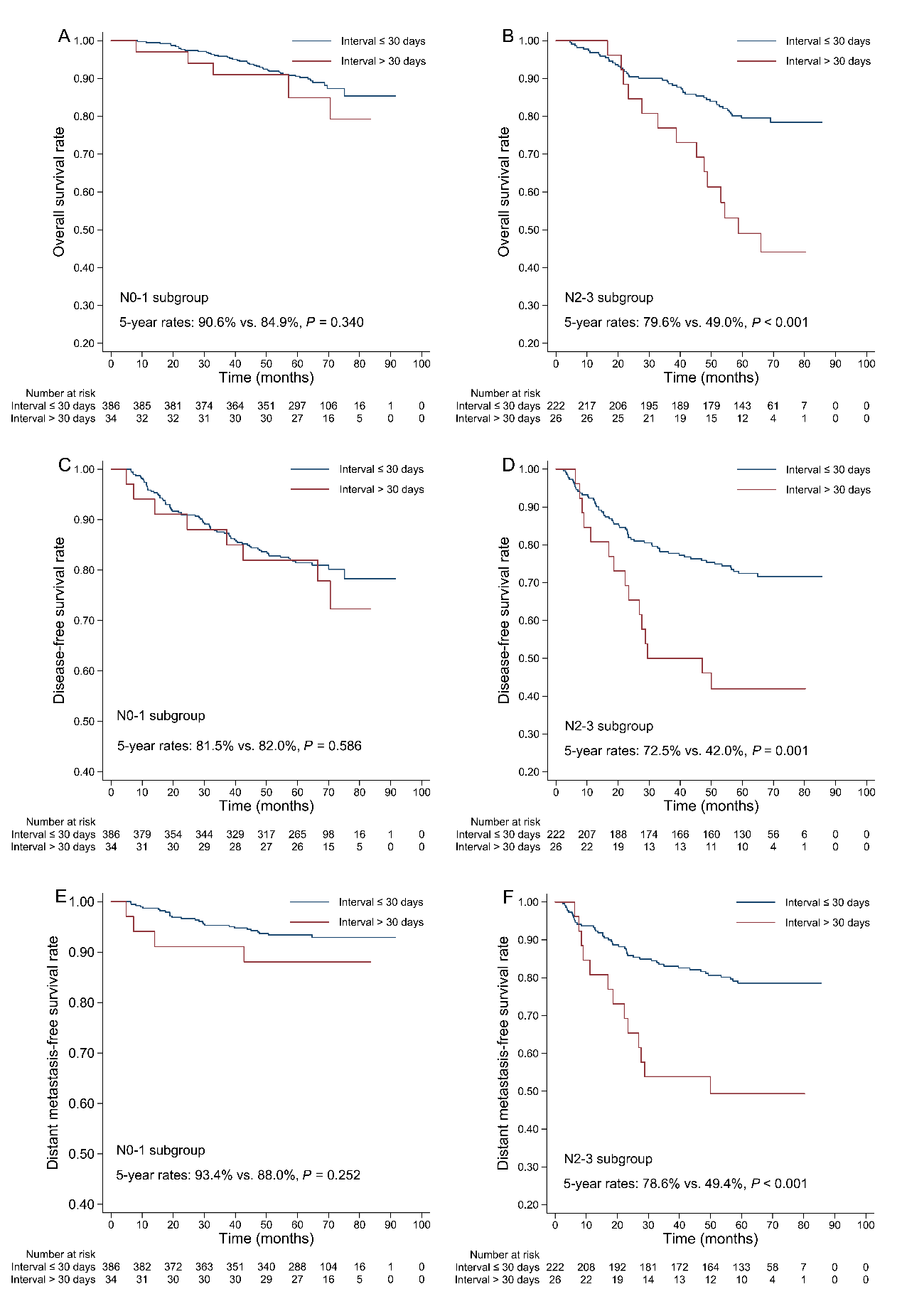


**Figure S3** Subgroup analyses based on N category. Kaplan–Meier survival curves were delineated based on the interval for overall survival (a, b), disease-free survival (c, d), distant metastasis-free survival (e, f) of N0-1 and N2-3 subgroups.


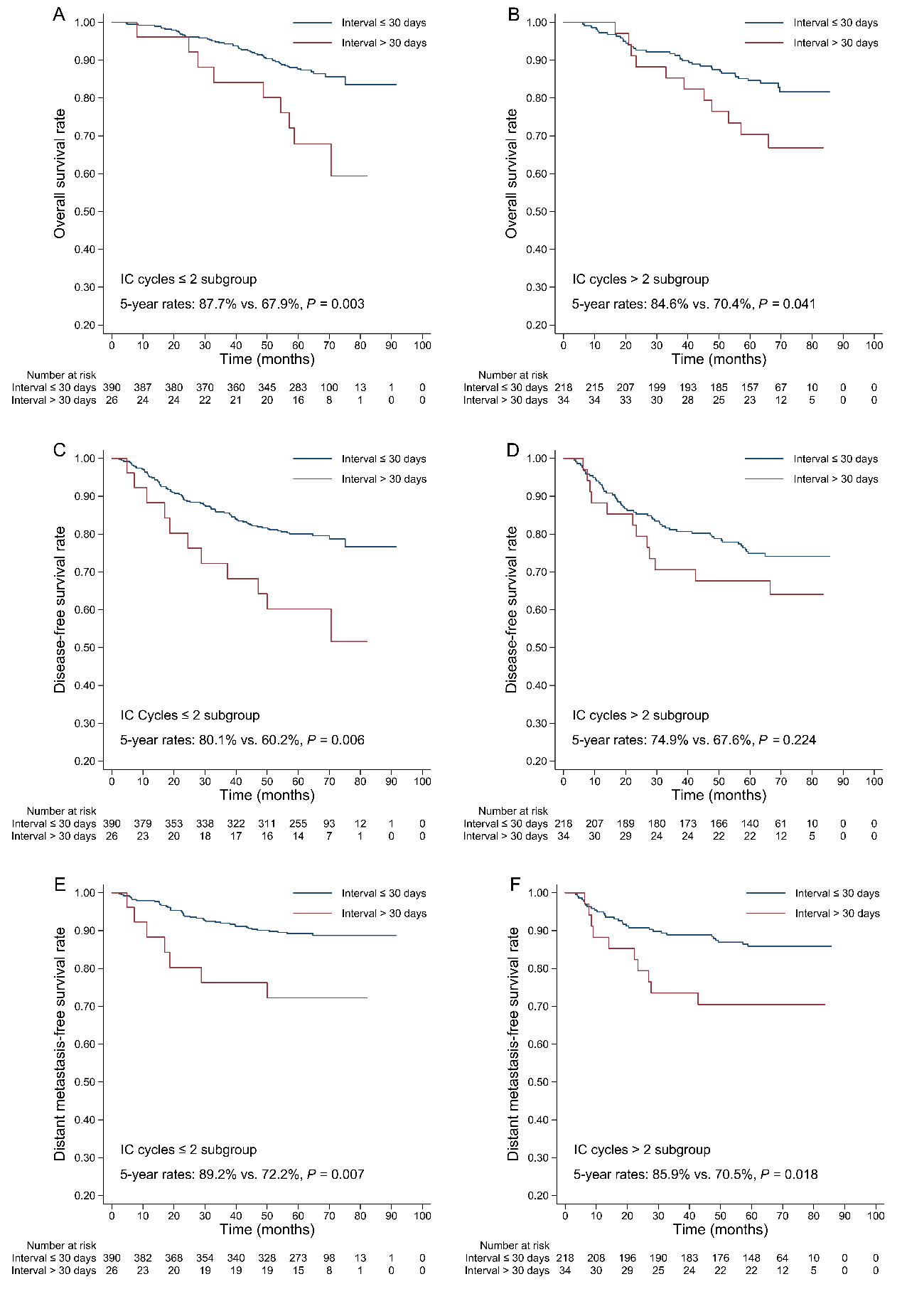


**Figure S4** Subgroup analyses based on IC cycles. Kaplan–Meier survival curves were delineated based on the interval for overall survival (a, b), disease-free survival (c, d), distant metastasis-free survival (e, f) of IC cycles ≤ 2 and > 2 subgroups. IC, induction chemotherapy.


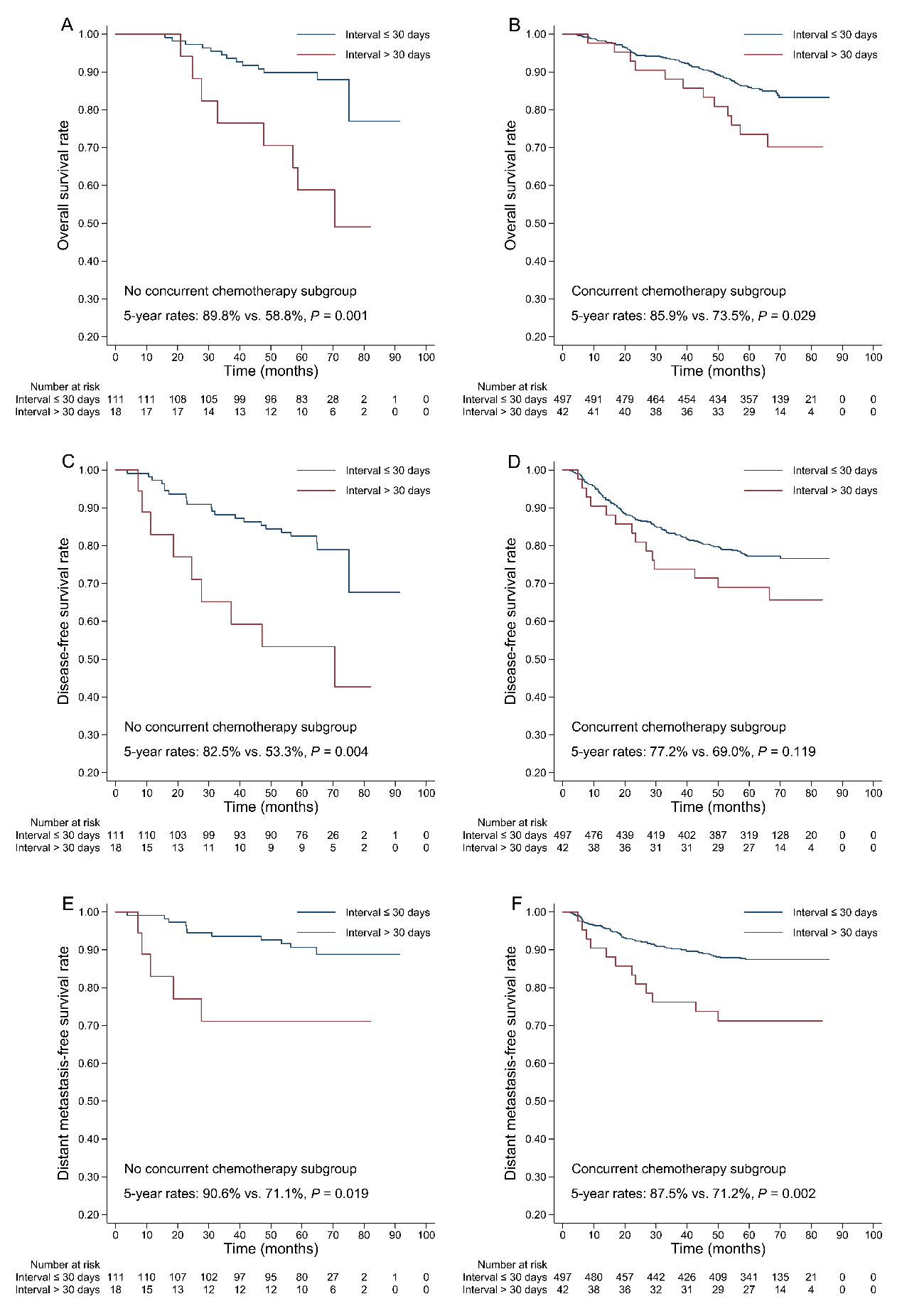


**Figure S5** Subgroup analyses based on concurrent chemotherapy. Kaplan–Meier survival curves were delineated based on the interval for overall survival (a, b), disease-free survival (c, d), distant metastasis-free survival (e, f) of concurrent chemotherapy and no concurrent chemotherapy subgroups.


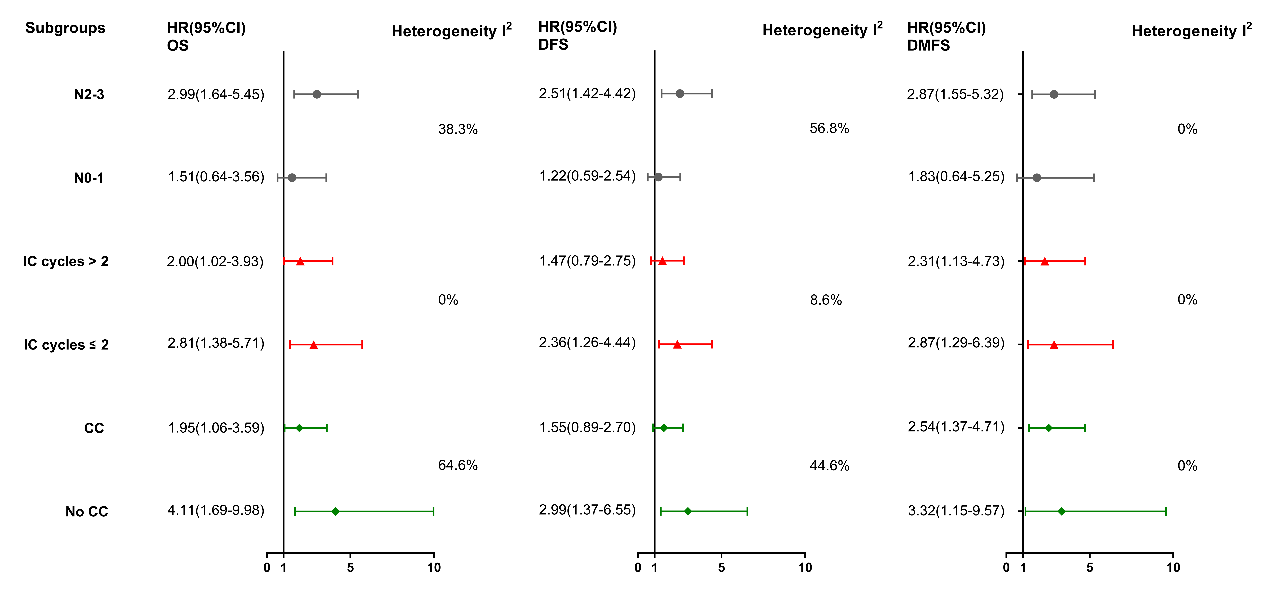


**Figure S6** Results of subgroup analyses summarized in forest plot. The HR (95% CI) of the interval for OS, DFS and DMFS in different subgroups and heterogeneity of HR between relative subgroups were shown. HR, hazard ratio; CI, confidence interval; OS, overall survival; DFS, disease-free survival; DMFS, distant metastasis-free survival; CC, concurrent chemotherapy.
