# Supplementary material for: The prolonged interval between induction chemotherapy and radiotherapy is associated with poor prognosis in patients with nasopharyngeal carcinoma
Source: Radiat Oncol. 2019 Jan 17;14:9. doi: 10.1186/s13014-019-1213-4 (PMC6335732; doi:10.1186/s13014-019-1213-4)
Supplement: Supplementary file 2 — Table S1. Baseline characteristics of included and excluded patients. (DOCX 19 kb) [file 13014_2019_1213_MOESM2_ESM.docx]

**Table S1.** Baseline characteristics of included and excluded patients.

| **Characteristics** | **All patients (2191)** | **Patients included (668)** | **IC patients without DNA data excluded (418)** |
| --- | --- | --- | --- |
| Age (years) | 45.6 ± 11.3 | 44.5 ± 11.0 | 46.1 ± 11.3 |
| Gender |  |  |  |
| Male | 1639 (74.8%) | 504 (75.4%) | 316 (75.6%) |
| Female | 552 (25.2%) | 164 (24.6%) | 102 (24.4%) |
| T category^*^ |  |  |  |
| T1 | 379 (17.3%) | 57 (8.5%) | 44 (10.5%) |
| T2 | 361 (16.5%) | 92 (13.8%) | 63 (15.1%) |
| T3 | 1036 (47.3%) | 326 (48.8%) | 198 (47.4%) |
| T4 | 415 (18.9%) | 193 (28.9%) | 113 (27.0%) |
| N category^*^ |  |  |  |
| N0 | 363 (16.6%) | 52 (7.8%) | 34 (8.1%) |
| N1 | 1219 (55.6%) | 368 (55.1%) | 223 (53.3%) |
| N2 | 313 (14.3%) | 108 (16.2%) | 76 (18.2%) |
| N3 | 296 (13.5%) | 140 (21.0%) | 85 (20.3%) |
| Stage^*^ |  |  |  |
| Ⅰ | 128 (5.8%) | 0 (0%) | 0 (0%) |
| II | 433 (19.8%) | 84 (12.6%) | 58 (13.9%) |
| III | 970 (44.3%) | 282 (42.2%) | 172 (41.1%) |
| IVa | 660 (30.1%) | 302 (45.2%) | 188 (45.0%) |
| WHO pathology type |  |  |  |
| Ⅰ | 12 (0.5%) | 6 (0.9%) | 2 (0.5%) |
| II | 100 (4.6%) | 32 (4.8%) | 16 (3.8%) |
| III | 2079 (94.9%) | 630 (94.3%) | 400 (95.7%) |

Data presented as number (%) or mean ± standard deviation.

Abbreviations: IC, induction chemotherapy.

^*^ According to the 8^th^ edition of AJCC/UICC staging system.
